# The “brain-gut” mechanism of postherpetic neuralgia: a mini-review

**DOI:** 10.3389/fneur.2025.1535136

**Published:** 2025-03-10

**Authors:** Peijun Zhang, Cuomaoji Zhang, Bixin Zheng, Yuntao Liu, Dingkun Zhang, Hong Xiao

**Affiliations:** ^1^Department of Pain Management, West China Hospital, Sichuan University, Chengdu, China; ^2^Department of Anesthesiology, Affiliated Sport Hospital of Chengdu Sport University, Chengdu Sport University, Chengdu, Sichuan, China; ^3^Laboratory of Clinical Proteomics and Metabolomics, Institutes for Systems Genetics, Frontiers Science Center for Disease-related Molecular Network, National Clinical Research Center for Geriatrics, West China Hospital, Sichuan University, Chengdu, China

**Keywords:** postherpetic neuralgia, brain-gut axis, mechanism, bacteria, microbiota, enteric nervous system

## Abstract

Postherpetic neuralgia (PHN), a representative type of neuropathic pain, has attracted much research on its diagnosis and therapy at the molecular level. Interestingly, this study based on the brain-gut axis provided a novel point of view to interpret the mechanism of PHN. Past neuroanatomical and neuroimaging studies of pain suggest that the prefrontal cortex, anterior cingulate cortex, amygdala, and other regions of the brain may play crucial roles in the descending inhibition of PHN. Dominant bacterial species in patients with PHN, such as Lactobacillus, generate short-chain fatty acids, including butyrate. Evidence indicates that disturbance of some metabolites (such as butyrate) is closely related to the development of hyperalgesia. In addition, tryptophan and 5-HT in the intestinal tract act as neurotransmitters that regulate the descending transmission of neuropathic pain signals. Concurrently, the enteric nervous system establishes close connections with the central nervous system through the vagus nerve and other pathways. This review aims to investigate and elucidate the molecular mechanisms associated with PHN, focusing on the interplay among PHN, the gut microbiota, and relevant metabolites while scrutinizing its pathogenesis.

## 1 Introduction

Postherpetic neuralgia (PHN) is defined as pain persisting for more than three months following the onset or healing of herpes zoster according to the International Association for the Study of Pain (IASP) among the latest classifications of chronic pain (1). The occurrence of herpes zoster is due to the reactivation of the Varicella Zoster Virus (VZV) that has been latent in the ganglion when immune function declines, leading to ganglioneuritis and hemorrhagic necrosis with associated neuritis, and even leptomeningitis and unilateral segmental poliomyelitis. Inflammatory damage to neurons is the cause of neuropathic pain. PHN is the state that results from the chronicization of this neuropathic pain (2, 3). As a typical refractory and debilitating form of neuropathic pain, PHN decreases the quality of life of patients (4, 5). The prevalence of PHN in adults has varied from 2.3% to 5% in China over the past four years (6, 7). A global epidemiology survey reported that the incidence of PHN in people over 50 years old is 18%. Among people over the age of 80, 33% are diagnosed (8). PHN manifests as persistent and refractory neuropathic pain, which has adverse effects on patients' physical and psychiatric health. Patients suffer from multiple types of pain, including constant deep, aching, or burning pain; paroxysmal, lancinating pain; hyperalgesia; and allodynia (9). In addition to intractable pain, patients with PHN often have comorbidities, such as insomnia, depression, or anxiety (10). Patients with long-term PHN appear to have a worse prognosis (11), which makes the complete eradication and elimination of PHN a considerable challenge. However, due to the complexity and individual differences of PHN, the mechanism underlying its chronicity remains unclear and has become a significant problem to be solved in PHN treatment. Therefore, it is necessary and urgent to explore the potential mechanism of PHN to better understand and guide future theranostic innovations and drug development.

The brain-gut axis, also called the gut-brain axis, microbiota-gut-brain axis, or brain-gut interaction, refers to bidirectional neurohumoral interactions among the brain, gut, and gut microbiome of the host (12, 13). Such bidirectional neurohumoural interactions always occur through neural and humoral pathways. Specifically, the neural pathways of the brain-gut axis include the enteric nervous system (ENS), vagus, sympathetic, and spinal nerves, while humoral pathways include cytokines, hormones, and neuropeptides (13, 14). In 1980s, researchers proposed the concept of the gut-brain axis based on the discovery that several polypeptide hormones originate from the gastroenteric tract and are present both in nerves and the brain (15). Previous studies have focused on the role of peptides such as cholecystokinin octapeptide, somatostatin, and bombesin in central and peripheral nerves (16) that coexist in the gut and brain. Comparatively, more researchers have focused on the types of molecules involved in brain-gut interactions (17, 18). The biological role of the brain-gut axis has been demonstrated in multiple disease models, including stress ulcers (19), functional dyspepsia (20), irritable bowel syndrome (21), inflammatory bowel diseases (22) [including Crohn's disease (23), ulcerative colitis (24)] and obesity (25). The results showed that stress is crucial for exacerbating gastrointestinal inflammation, reducing immunity, and increasing digestive tract vulnerability (22). In addition, researchers have explored potential therapeutic mechanisms based on the brain-gut axis (26, 27), such as monitoring the neurological and etiological effects of ingesting specific strains of bacteria (28, 29). Psychotropic drugs have been proposed for use in treating digestive diseases such as irritable bowel syndrome (27). In the last decade, scientists have investigated the relationships between the brain-gut axis and neuropsychiatric illnesses such as autism (30), anxiety, fear (31), bipolar disorder (32), depression, anxiety (33, 34), obsessive-compulsive disorder (35) and neurodegenerative diseases (36) (including Parkinson's disease (37), Alzheimer's disease (38) and central nervous system demyelination (39)). The diversity of models involved in the above brain-gut axis studies needs to be improved. Research on the nervous system in the brain-gut axis has focused mainly on psychiatric rather than neurological diseases.

Interestingly, recent studies revealed that the brain-gut axis is involved in the development of visceral hypersensitivity and pain (40–42), especially in individuals with irritable bowel syndrome (43). Neural sensitization may be driven by peripheral mechanisms within the intestinal wall, encompassing an interplay between immunocytes, enterochromaffin cells, resident macrophages, neurons, and smooth muscles. In the brain, neuronal synaptic changes and enhanced release of neurotransmitters in the spinal cord and brain lead to central sensitization (44). Alterations in the brain-gut axis originating from small intestinal bacterial overgrowth or subclinical intestinal infections such as giardiasis may be significant causes of cormobidity of fibromyalgia and irritable bowel syndrome (45). Several therapeutic approaches have been suggested for the brain-gut axis, such as dietary modifications, oral probiotics, antibiotics, and anti-inflammatory agents (46, 47). These therapeutic approaches have shown some efficacy in gastrointestinal diseases, but their effects under other conditions still need to be clarified. Alternatively, developing the brain-gut axis theory provides a new method for managing refractory pain. Peripheral pain from both visceral and somatosensory nerves is transmitted to the brain via spinal dorsal horn neurons (48, 49). Therefore, it is reasonable to speculate that the brain-gut axis is involved in the occurrence and development of neuropathic pain, such as PHN, to some extent. Potential therapeutic methodologies based on the brain-gut axis are expected to be used to treat PHN and improve patients' quality of life.

In this review, we collected papers published in PHN-related fields over the last 20 years to explore and summarize PHN-related molecular mechanisms among PHN, the gut microbiota, and relevant metabolites. And we examined the pathogenesis and development of PHN from the perspective of the brain-gut axis and identified potential targets for improvement of gastrointestinal motility treatment of PHN in the future.

## 2 Methods

We conducted searches using the keywords “pain”, “neuropathic pain”, “postherpetic neuralgia”, “pathway”, “mechanism”, and “fMRI” to find articles concerning the involvement of the brain in PHN. Similarly, we utilized the keywords “intestinal”, “gut”, “bacterium”, “microorganism”, and “postherpetic neuralgia” to explore research on the intestinal microbiota of PHN patients. Based on the predominant bacterial species suggested by relevant studies, we searched for the relationship between their metabolites and pain modulation. Using “brain-gut axis” and “brain-gut interaction” as keywords, we searched for the main mechanisms and pathways confirmed in current research, further exploring the bidirectional effects of PHN on the intestine and brain.

## 3 Recent findings

### 3.1 The role of the brain in PHN

As the body's most complex nervous system organ, the brain perceives pain, directing the body's response. Regardless of whether the pain originates from herpes zoster, patients with various types of pain show high similarity in pain transduction pathways. The spinothalamic tract projects the stimulation it receives to the ventroposterolateral nucleus of the contralateral thalamus. Superior neurons transmit pain signals to relevant somatosensory cortical regions, including the primary somatosensory cortex, secondary somatosensory cortex, posterior insula, medial parietal operculum, orbitofrontal cortex, dorsal-lateral prefrontal cortex, extended amygdala, and cingulate cortex. Parts of pain signals are transferred between the anterior and posterior insular cortex. The final pain experience combines sensory input and behavioral and cognitive interpretations of pain (50–52). The pathway is regulated by the descending modulation system, which descends from the brain to the spinal cord and modifies incoming somatosensory information to alter the perception and reactions to somatosensory stimuli, resulting in increased or decreased pain (5). The descending inhibitory pathway consists of the amygdala, the prefrontal cortex, the periaqueductal gray (PAG), and the rostral ventromedial medulla (RVM) from top to bottom (53). Finally, the RVM sends diffuse bilateral projections to the dorsal horn, terminating at multiple levels (54). Here, we list a few brain structural regions related to pain (Supplementary Tables S1, S2).

PHN has unique characteristics of the central nervous system. Some functional imaging studies conducted on PHN patients have shown significant changes in gray matter volume, functional connections, regional homogeneity (ReHo), fractional aptitude of low-frequency fluctuation (fALFF), mean diffusivity (MD), and other parameters in multiple brain regions (such as the temporal lobe, insula, frontal lobe, cingulate gyrus, amygdala, and thalamus) compared with those in healthy controls (Supplementary Table S3). However, there are significant differences between the results of different studies, possibly because of the following reasons: 1. The affected side was not classified; 2. There is a lack of extensive sample size studies; 3. Differences in instruments and equipment. Since the completion of resting-state fMRI requires the subject to stop thinking activities, stay awake, and keep the head still, good cooperation is required during the examination. The above factors will confound the research results if the combination is not good. In addition, a study has shown that neurovascular aging can affect fMRI imaging in some aspects (55). Furthermore, functional changes in brain regions on fMRI can only indicate that they are relay stations in the pain transmission pathway and cannot clarify the signaling pathways and regulatory mechanisms involved. However, further primary studies are needed to verify its role in pain transduction pathways.

In summary, within the central nervous system of PHN patients, the thalamus is responsible for the ascending transmission of pain information; the somatosensory cortex is involved in pain perception; the insular cortex is like a relay station of pain signal and related to pain hypersensitivity (56); the ACC, PFC, and amygdala contribute to the formation of unpleasant emotions; and the PAG and RVM participate in the descending inhibition of pain signals. The specific roles of other cortical areas, the precuneus, and the brainstem in PHN require further investigation.

### 3.2 The relationship between the gut microenvironment and PHN

Research indicates that inflammatory bowel disease can increase the risk of developing herpes zoster (57). Dysregulation of the immune response of the gut microbiota may be involved in the development of postherpetic neuralgia (58). However, there is only one study on the gut microbiota in patients with PHN (59). Since there is a need for more relevant studies in other regions, its generalizability is controversial. Therefore, based on the limited research results, we speculate on categorizing predominant gut bacteria in patients with PHN and healthy controls.

As a major resident bacterium in the human gut, *Lactobacillus* breaks down intestinal polysaccharides and starch into glucose and maltose. This process provides substrates for bacteria lacking this function, such as *Eubacterium ramulus*, indirectly promoting the production of butyrate (60) As a short-chain fatty acid (SCFA), butyrate may relieve neuropathic pain in obese mice by regulating gene expression and immune function in the peripheral nervous system (61). Its immunomodulatory effects may be achieved by suppressing proinflammatory cytokines (62). In an animal model of chronic compression injury, repeated administration of butyrate alleviated mechanical and thermal hyperalgesia, thereby mitigating neuropathic pain (63). One study suggested that butyrate alleviates neuropathic pain by increasing withdrawal thresholds and reducing hyperalgesia (64). This may be achieved through the induction of regulatory T cells, the reduction of proinflammatory cytokines, and the downregulation of Toll-like receptor (TLR) 4 receptors (65).

The *Lachnospiraceae* family is a significant branch of the *Firmicutes* phylum, which, along with the *Bacteroidetes* phylum, comprises the two most crucial microbial groups in the human gut. *Lachnospiraceae* species breakdown starch and various sugars, generating SCFAs. The *Roseburia* species primarily synthesizes butyrate as the primary SCFA, in addition to acetate (66). Supplementation with *Roseburia hominis* significantly increased cecal butyrate levels, alleviated visceral hypersensitivity and prevented the decreased expression of occludin in the colon of rats (67). In patients with PHN, there is a decrease in the abundance of bacterial species that produce butyrate in the gut (59). However, the underlying mechanism remains unknown, suggesting butyrate may take part in endogenous analgesia.

*Clostridium* in the gut is an anaerobic bacterium, with only a few species being pathogenic. It can form endospores and has extensive applications in medical and bioengineering fields. For instance, it can be utilized for treating tumors and antibiotic-associated diarrhea. Some species within *Clostridium* can generate acetate, propionate, butyrate, butanol, acetone, formate, and other compounds through various metabolic pathways. Hence, they are utilized to produce organic solvents (68, 69). *Clostridia* sourced from humans can boost the population and efficacy of colonic Treg cells (a specialized subpopulation of T cells that could suppress immune responses to keep immunological homeostasis (70)) in colonized rodents, consequently reducing symptoms of experimental allergic diarrhea and colitis (71). Research indicates that the dysregulation of Treg cells and effector T cells (which could secrete interleukin 1 or 17) can lead to an overly active immune response and elevated levels of neurological inflammation and can promote the progression of conditions such as neuropathic pain disorders. PHN represents a classic form of peripheral neuropathic pain, from which we can infer that an increase in gut Treg cells contributes to the advancement of this condition (72).

In conclusion, predominant bacterial species in the gut of PHN patients may regulate the levels of neuroinflammation, enhance pain thresholds, and alleviate tactile hypersensitivity to some extent by participating in the synthesis of short-chain fatty acids, thereby modulating the quantity of T-cell subsets.

### 3.3 The molecular basis of brain-gut interaction

Due to the exclusive tropism of the varicella-zoster virus for humans, many rodent-based PHN models currently fail to accurately reflect authentic pathological and physiological conditions. The lack of researches focused on the gut-brain mechanism of PHN in humans leads to many mechanisms being speculative. Current mainstream studies have shown that the gut-brain interaction is primarily manifested in the following aspects: the connection between the enteric nervous system and the central nervous system and the impact of gut metabolites with neurotransmitter or neuromodulator properties on the central nervous system (73).

The ENS spans from the upper esophagus to the internal anal sphincter, establishing connections with the biliary tract, liver, gallbladder, and pancreas. The input and output of ENS signals are primarily mediated through the vagus nerve and splanchnic nerves. Research has shown that the vagal neural crest of mice enters the foregut at approximately embryonic days 7-9.5. The majority of the ENS originates from the vagal neural crest, with a smaller portion originating from the sacral neural crest (74). The vagus nerve collects mechanical and chemical sensations from the intestine, influencing the movement of smooth muscles. Splanchnic nerves can transmit pain signals and regulate glucose metabolism. The ENS can be divided into two neural plexuses: the myenteric plexus and the submucosal plexus. The myenteric plexus is situated between the smooth muscles of the intestine and governs the movement of the intestinal smooth muscles. The submucosal plexus is located between the mucosa and smooth muscle of the intestines, dominating intestinal absorption and secretion and comprising various types of neurons, including intrinsic primary afferent neurons, motor neurons, enteric glia, and interneurons (75, 76).

Vagus nerve stimulation (VNS) is a technique that relies on surgical implantation, where electrodes are placed on the surface of the vagus nerve, and a pulse generator connected to it is implanted under the skin. The pulse generator releases pulsed electrical currents to control epileptic seizures (77). VNS was once considered an adjunctive treatment for patients aged four years and older with medically refractory partial-onset seizures (78). Moreover, the medication pregabalin, used to treat neuropathic pain, including PHN, is also an antiepileptic drug. Therefore, we have reason to suspect a potential connection between the vagus nerve and neuropathic pain, including PHN. Intermittent vagus nerve stimulation enhances the expression of neuronal fos in the medullary vagal complex, locus coeruleus, and various thalamic and hypothalamic nuclei Fos serves as an indicator of heightened metabolic activity. Additionally, VNS leads to the upregulation of brain-derived neurotrophic factor and fibroblast growth factor in the hippocampus and cerebral cortex, a reduction in nerve growth factor mRNA abundance in the hippocampus, and an increase in the norepinephrine concentration in the prefrontal cortex (79). Pregabalin binds to the α_2_ − δ subunit of voltage-gated calcium channels on the neocortex, amygdala, hippocampus, striatum, dorsal horn of the spinal cord, cerebellum, and habenula, reducing the influx of calcium ions into cells. Pregabalin can decrease the release of glutamate and norepinephrine in the trigeminal nucleus and neocortex while simultaneously reducing synaptic potentials and intrasynaptic currents (80). This appears to contradict the effects of VNS. Currently, there is a lack of direct research on the functionality of the vagus nerve, neuropathic pain, and antiepileptic drugs, perhaps suggesting a potential avenue for future investigations.

Tryptophan is an essential amino acid in the human body that is absorbed through food intake and is primarily metabolized via various pathways: 1. Conversion to kynurenine in the liver, 2. Transformation to 5-HT in enterochromaffin cells in the intestine; 3. Iron is converted to indole under the influence of intestinal microorganisms. Kynurenine mainly affects the immune system. Kynurenine regulates colonic epithelial cell immunity by activating colonic aryl hydrocarbon receptors. It actively enters the central nervous system and is metabolized into kynurenic acid, quinolinic acid, and anthranilic acid. Kynurenic acid is an antagonist of N-methyl-D-aspartate (NMDA) receptors in the CNS. Additionally, it opposes nicotinic α−7 receptors expressed on macrophages and lymphocytes, thereby increasing the release of TNFα from macrophages in the reticuloendothelial system (81). L-4-Chlorokynurenine attenuates overactive glutamatergic transmission via NMDA receptors by blocking the allosteric glycine B coagonist site on the receptor (82). L-4-Chlorokynurenine inhibits central sensitization, making it a potential therapeutic option for neuropathic pain, including PHN.

The regulation of pain by 5-HT is comprehensive. 5-HT is secreted by enterochromaffin cells on the intestinal surface and released into the submucosa to act on intrinsic serotonergic neurons in the submucosal plexus of the ENS (81). Specifically, upon receiving information about intestinal distension, enterochromaffin cells secrete 5-HT, activating 5-HT_3_ and 5-HT_4_ receptors on submucosal splanchnic nerves and the vagus nerve. Subsequently, these signals ascend through the dorsal horn of the spinal cord, conveying nociceptive signals to the thalamus and projecting to the cortex. In this process, 5-HT participates in hyperalgesia through the mediation of 5-HT_1_ and 5-HT_2_ receptors in the spinal cord, a process that is regulated by the serotonin transporter (83). 5-HT_1A_ receptors is one of six subtypes of 5-HT_1_ receptors, a large number of distributions in raphe nucleus, amygdala, cingulate cortex, insula, prefrontal cortex and spinal dorsal horn. The 5-HT_1A_ receptor functions as an inhibitory autoreceptor, presynaptically suppressing nociceptive transmission into laminae I and II of the dorsal horn. This effect is mediated by the upregulation and downregulation of 5-HT_1A_ receptors in the rostral ventromedial medulla. 5-HT_2_ receptor is coupled to the G q/11 protein. And 5-HT_2_ receptors which are located on peripheral neurons induce primary thermal hyperalgesia and secondary mechanical allodynia (84). 5-HT_3_ receptor is a ligand-gated cation channel.Antagonists of 5-HT_3_ receptors can block nonspecific ion channels, restricting the influx of Na^+^ and Ca^2+^ and efflux of Cl^−^, thereby interrupting the transmission of pain signals and mitigating visceral hypersensitivity to some extent (85). A portion of the 5-HT produced in the gut enters the bloodstream and is absorbed and stored by platelets. In the central nervous system, 5-HTergic neurons originating from the nucleus raphe magnus regulate the descending transmission of neuropathic pain signals, including PHN. Among these receptors, the 5-HT_3_ receptor couples with cAMP (cyclic adenosine monophosphate), enhancing nociceptive sensations, tactile allodynia and thermal hyperalgesia. While the 5-HT_7_ receptor can increase the release of endorphins or GABA (gamma-amino-butyric acid), exerting a pain-relieving effect. It also plays a critical role in opiate-induced antinociception, probably through activation of descending inhibition (86, 87).The administration of systemic antidepressants such as selective serotonin reuptake inhibitors (SSRIs) can increase the concentration of 5-HT in the spinal cord, producing anti-hypersensitive effects in rodent models of neuropathic pain (88). Studies have indicated that 5-HT receptor subtypes play distinct roles in neuropathic pain. 5-HT_2A_ and 5-HT_4_ may promote neuropathic pain, while 5-HT_1A_, 5-HT_1B/1D_, 5-HT_2C_, and 5-HT_7_ may exert inhibitory effects. The role of 5-HT_3_ in neuropathic pain could be dual (89, 90). In fact, the actions of these receptors are likely to be synergistic. The summary of the roles of 5-HT receptors in neuropathic pain does not account for all research findings. Several factors, such as the specific cause of neuropathic pain and the concentration of 5-HT in the body, may influence which receptor plays a dominant role.

**Figure 1:**
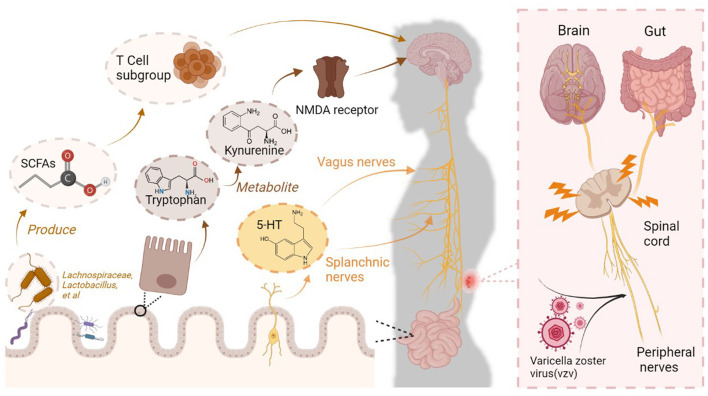
Complex connections between the brain and intestine in PHN patients. Varicella zoster virus (VZV) invades and damages peripheral nerves, ascending to the brain through the spinal cord. Central sensitization in the brain contributes to PHN becoming a refractory condition. This process is accompanied by changes in gut microbiota colonization, which involves mechanisms for responding to pain. Specific bacteria in the gut produce short-chain fatty acids (SCFAs), which regulate the level of neuroinflammation by modulating T-cell subsets, thereby adjusting pain sensitivity. The enteric nervous system (ENS) receives neurotransmitters such as 5-HT, which transmit nociceptive signals to the central nervous system via the vagus and splanchnic nerves. The gut metabolite kynurenine enters the central nervous system via the circulation and acts as an N-methyl-D-aspartate (NMDA) receptor antagonist, exerting analgesic effects.

Gut and brain interactions manifest in multiple aspects and at multiple levels (Figure 1). In PHN patients, pain is modulated through the ENS and intestinal metabolites, while the disease also affects inflammation levels in the nervous system and intestinal metabolite levels. However, it must be noted that this interaction is complex and subject to individual differences. The specific underlying mechanisms require further research.

## 4 Limitations

As mentioned earlier, due to the specific infection of VZV in humans, it is impossible to replicate patients' pathological and physiological states in rodent models. Clinical studies based on patient data also have certain limitations. Further in-depth research requires support from primary experimental results. Consequently, research on PHN is subject to certain limitations. PHN is the only type of neuropathic pain involving one to two peripheral nerve segments caused by viral infection. However, in many studies on the central mechanisms of neuropathic pain, PHN has yet to be studied separately. The generalizability of new drugs developed based on related research on neuropathic pain in PHN patients is unknown. Therefore, it is necessary to conduct large-scale studies on neuroimaging, neurophysiology, neuroimmunology, and other related aspects of PHN to fill the gaps in this field. Currently, more research on the gut microbiota of PHN patients is needed, leading to a suboptimal representation of the microbiological characteristics of patients' intestines. Additionally, there is a lack of mechanistic studies on the interaction between the gut and central nervous system in PHN patients. Many of the mechanisms mentioned in the current literature are speculative and derived from neuropathic pain-based hypotheses, and their validity needs further experimental design and research.

## 5 Conclusion and prospects

This article elucidated the brain-gut regulatory mechanisms in PHN patients by reviewing the pathogenic mechanisms, neurophysiology, neuroimaging, and dominant bacterial strains associated with PHN. At the brain level, the thalamus is responsible for the ascending transmission of pain information in PHN patients; the somatosensory cortex is involved in pain perception; the ACC, PFC, and amygdala contribute to the formation of unpleasant emotions; and the PAG and RVM exert descending inhibition on pain signals. At the gut level, predominant bacterial species in the gut of PHN patients may regulate the levels of neuroinflammation, enhance pain thresholds, and alleviate tactile hypersensitivity to some extent by participating in the synthesis of short-chain fatty acids, thereby modulating the quantity of T-cell subsets. A study on PHN at the brain-gut level revealed that interactions between the gut and the brain manifest at multiple aspects and levels. In PHN patients, pain is modulated through the ENS and intestinal metabolites, while the disease also affects inflammation levels in the nervous system and intestinal metabolite levels. Although this review has certain limitations in that the role of other brain regions and bacteria in PHN lacks satisfactory interpretation due to insufficient supplementation of existing works, this review could provide a theoretical basis for proposing new treatment methods and direct future research endeavors toward PHN. Future studies investigating the brain-gut mechanisms of PHN could provide potential therapeutic strategies for disease management and improvement of patient quality of life.
