# Supplementary material for: The “brain-gut” mechanism of postherpetic neuralgia: a mini-review
Source: Front Neurol. 2025 Mar 10;16:1535136. doi: 10.3389/fneur.2025.1535136 (PMC11932021; doi:10.3389/fneur.2025.1535136)
Supplement: Supplementary file 1 [file Presentation_1.pdf]

Supplementary Material for The "brain-gut" mechanism of Postherpetic Neuralgia: a mini-review.

1 FIGURE S1.COMPLEX CONNECTIONS BETWEEN THE BRAIN AND INTESTINE IN PHN PATIENTS.

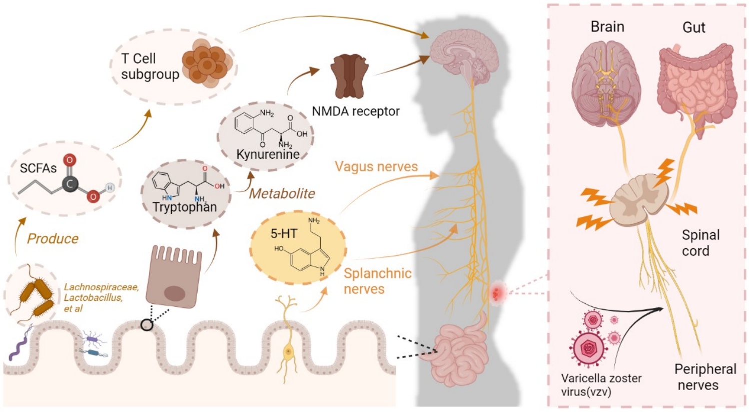

Figure S1. Complex connections between the brain and intestine in PHN patients. Varicella zoster virus (VZV) invades and damages peripheral nerves, ascending to the brain through the spinal cord. Central sensitization in the brain contributes to PHN becoming a refractory condition. This process is accompanied by changes in gut microbiota colonization, which involves mechanisms for responding to pain. Specific bacteria in the gut produce short-chain fatty acids (SCFAs), which regulate the level of neuroinflammation by modulating T-cell subsets, thereby adjusting pain sensitivity. The enteric nervous system (ENS) receives neurotransmitters such as 5-HT, which transmit nociceptive signals to the central nervous system via the vagus and splanchnic nerves. The gut metabolite kynurenine enters the central nervous system via the circulation and acts as an N-methyl-D-aspartate (NMDA) receptor antagonist, exerting analgesic effects.

2 TABLE S1,S2. THE FUNCTION OF BRAIN REGIONS IN THE PATHWAY OF PAIN

3 TABLE S3. FMRI IMAGING CHARACTERISTICS OF THE PHN/HERPETIC ZOSTER IN DIFFERENT BRAIN REGIONS

Table S1. The function of brain regions in the pathway of pain

Brain structural regions	Abbreviation	Role of pain pathways	Citations
Primary somatosensory cortex	SI	Located in the postcentral gyrus, Brodmann 1, 2, 3a, 3b, critical point of the ascending transmission of nociceptive sensations	Vierck et al. (2013)
secondary somatosensory cortex	SII	Receive fibers originated from SI, located in the region of the parietal operculum,in the upper bank of the lateral sulcus, can respond to somatosensory and visual stimuli, maybe related to self-consciousness and self-location, social relations, whole body image and self-recognition, and metaphoric extrapolations	Bretas et al. (2020)
anterior cingulate cortex	ACC	a key region of the limbic system, involved in cognitive and emotional processing, has functions of conflict monitoring, error detection, attention,and reward-based learning and anticipation,receives nociceptive signals, and sends descending signals to the nucleus accumbens and rostral ventromedial medulla, then eventually modulates the pain sensation from spinal nociception	Xiao et al. (2021)
prefrontal cortex	PFC	can be divided into several multifunctional parts,It is reported that the dorsolateral prefrontal cortex (DLPFC) has the function of suppression of pain , maintenance of pain inhibition and alleviation of pain related unpleasantness.Chronic pain is associated with decreased grey matter volume in DLPFC.The bilateral medial prefrontal cortex receives projections from the locus coeruleus, DLPFC, hippocampus, ventral tegmental area, insular cortex directly, while mainly projecting to nucleus accumbens and periaqueductal grey.PFC is reciprocally connected to amygdala	Seminowicz and Moayedi (2017); Ong et al. (2019)
amygdala		A part of limbic system. plays an important role in emotional-affective aspects of pain.Made up of basolateral complex(BLA), the central nucleus (CeA) and the intercalated cell clusters (ITC). input signals from cortex and thalamus and output to brainstem, hypothalamus and basal forebrain regions	Neugebauer et al. (2020)
periaqueductal gray	PAG	A critical relay station of descending inhibitory circuit,integrates information from cortical and subcortical areas, stimulation of PAG induces release of endogenous opioids	Bagley and Ingram (2020)

Table S2. Continued Table of Table S1

Brain structural regions	Abbreviation	Role of pain pathways	Citations
Rostral ventromedial medulla	RVM	Centered on the nucleus raphe magnus with adjacent ventromedial reticular formation. Receives top-down input from higher structures via PAG. Receives bottom-up input of nociceptive information. Direct actions in the RVM partly mediate analgesic effects of mu-opioid agonists. Functions to facilitate as well as inhibit pain	Chen and Heinricher (2022)

Table S3. fMRI imaging characteristics of the PHN/herpetic zoster in different brain regions

Area of brain	Image feature in PHN/herpetic zoster	Citations
Temporal cortex	less gray matter volume in T1-weight image,decrease in ReHo and fALFF in resting-state functional MRI,decreased fractional anisotropy (FA) in temporal lobe, decreased mean diffusivity (MD) in right superior temporal lobe, decreased ALFF in left superior temporal gyrus, increased ALFF in the left inferior temporal gyrus and right middle temporal gyrus in a combined DTI and rs-fMRI	Qiu et al. (2021); Cao et al. (2017); Dai et al. (2020)
Insula cortex	less gray matter volume in T1-weight image, decreased ALFF in right insula in a combined DTI and rs-fMRI	Qiu et al. (2021); Dai et al. (2020)
fusiform cortex	less gray matter volume in T1-weight image	Qiu et al. (2021)
precuneus	decreased mean diffusivity (MD) in left precuneus in a combined DTI and rs-fMRI	Dai et al. (2020)
frontal cortex	weaker morphological connectivity with right precuneus in left opercular part of inferior frontal gyrus, and left orbital part of middle frontal gyrus shows a stronger degree in T1-weight image, decreased functional connectivity with PAG in rs-fMRI	Qiu et al. (2021); Li et al. (2020)
anterior cingulate cortex	left anterior cingulate gyrus has a weaker degree in T1-weight image,decreased FA in the brain area of left anterior cingulate gyrus in a combined DTI and rs-fMRI,decreased gray matter volume in rs-fMRI	Qiu et al. (2021); Dai et al. (2020); Tang et al. (2021)
Amygdala	Smaller gray matter volumes in fMRI	Li et al. (2020)
Thalamus	Larger gray matter volumes in right thalamus in fMRI, while another research holds opposite view and shows that PHN patients has increased functional connectivity between the thalamus and somatosensory cortices	Li et al. (2020); Tang et al. (2021)
Brain stem	decreased ALFF in the right part of brainstem	Dai et al. (2020)

REFERENCES

Bagley, E. E. and Ingram, S. L. (2020). Endogenous opioid peptides in the descending pain modulatory circuit. *Neuropharmacology* 173, 108131. doi:10.1016/j.neuropharm.2020.108131

Bretas, R. V., Taoka, M., Suzuki, H., and Iriki, A. (2020). Secondary somatosensory cortex of primates: beyond body maps, toward conscious self-in-the-world maps. *Exp Brain Res* 238, 259–272. doi:10.1007/s00221-020-05727-9

Cao, S., Li, Y., Deng, W., Qin, B., Zhang, Y., Xie, P., et al. (2017). Local brain activity differences between herpes zoster and postherpetic neuralgia patients: A resting-state functional mri study. *Pain Physician* 20, E687–e699

Chen, Q. and Heinricher, M. M. (2022). Shifting the balance: How top-down and bottom-up input modulate pain via the rostral ventromedial medulla. *Front Pain Res (Lausanne)* 3, 932476. doi:10.3389/fpain.2022.932476

Dai, H., Jiang, C., Wu, G., Huang, R., Jin, X., Zhang, Z., et al. (2020). A combined dti and resting state functional mri study in patients with postherpetic neuralgia. *Jpn J Radiol* 38, 440–450. doi:10.1007/s11604-020-00926-4

Li, H., Li, X., Feng, Y., Gao, F., Kong, Y., and Hu, L. (2020). Deficits in ascending and descending pain modulation pathways in patients with postherpetic neuralgia. *Neuroimage* 221, 117186. doi:10.1016/j.neuroimage.2020.117186

Neugebauer, V., Mazzitelli, M., Cragg, B., Ji, G., Navratilova, E., and Porreca, F. (2020). Amygdala, neuropeptides, and chronic pain-related affective behaviors. *Neuropharmacology* 170, 108052. doi:10.1016/j.neuropharm.2020.108052

Ong, W. Y., Stohler, C. S., and Herr, D. R. (2019). Role of the prefrontal cortex in pain processing. *Mol Neurobiol* 56, 1137–1166. doi:10.1007/s12035-018-1130-9

Qiu, J., Du, M., Yang, J., Lin, Z., Qin, N., Sun, X., et al. (2021). The brain's structural differences between postherpetic neuralgia and lower back pain. *Sci Rep* 11, 22455. doi:10.1038/s41598-021-01915-x

Seminowicz, D. A. and Moayedi, M. (2017). The dorsolateral prefrontal cortex in acute and chronic pain. *J Pain* 18, 1027–1035. doi:10.1016/j.jpain.2017.03.008

Tang, Y., Ren, C., Wang, M., Dai, G., Xiao, Y., Wang, S., et al. (2021). Altered gray matter volume and functional connectivity in patients with herpes zoster and postherpetic neuralgia. *Brain Res* 1769, 147608. doi:10.1016/j.brainres.2021.147608

Vierck, C. J., Whitsel, B. L., Favorov, O. V., Brown, A. W., and Tommerdahl, M. (2013). Role of primary somatosensory cortex in the coding of pain. *Pain* 154, 334–344. doi:10.1016/j.pain.2012.10.021

Xiao, X., Ding, M., and Zhang, Y. Q. (2021). Role of the anterior cingulate cortex in translational pain research. *Neurosci Bull* 37, 405–422. doi:10.1007/s12264-020-00615-2
